# Irisin attenuates astrocyte activation and rescues memory in Alzheimer model mice

**DOI:** 10.1002/alz.091904

**Published:** 2025-01-03

**Authors:** Luana Heimfarth, Felipe C Ribeiro, Juliana Fortuna, Sergio T Ferreira

**Affiliations:** ^1^ Federal University of Rio de Janeiro, Rio de Janeiro, Rio de Janeiro Brazil; ^2^ Federal University of Rio de Janeiro, Rio de Janeiro Brazil

## Abstract

**Background:**

Alzheimer’s disease (AD) is a progressive neurodegenerative disease characterized by synapse and memory failure, and severe cognitive impairment. Physical exercise stimulates neuroprotective pathways, has pro‐cognitive actions, and has been reported to alleviate memory impairment in AD. Irisin, an exercise‐induced hormone, is secreted following proteolytic cleavage of fibronectin type‐III‐domain‐containing 5 (FNDC5). Irisin regulates peripheral metabolism, and has been found to protect synapses and rescue memory in mouse models of AD. The aim of the current study was to evaluate the possible role of irisin in astrocytes within the framework of AD pathophysiology.

**Methods:**

We exposed primary cortical astrocyte cultures to irisin (25 nM), and the expression of neurotrophic factors and/or MAPK activity were determined by RT‐PCR or western blot, respectively. Cognitive impairment was investigated in C57BL/6 mice that received an intracerebroventricular (i.c.v.) infusion of AβOs in the presence or absence of irisin.

**Result:**

We demonstrate that irisin promotes the expression of brain‐derived neurotrophic factor (BDNF) and glial cell line‐derived neurotrophic factor (GDNF) in astrocytes, and stimulates transient activation of extracellular signal‐regulated kinase 1/2 (ERK 1/2). We further show that irisin attenuates memory impairments in an AD mouse model, and modulates GDNF and insulin‐like growth factor‐1 (IGF1) signaling *in vivo*.

**Conclusion:**

Our findings support the idea that physiological protection by irisin against synaptotoxic AβOs can be mediated by modulation of astrocyte‐derived factors.

